# Gene expression profiling upon ^212^Pb-TCMC-trastuzumab treatment in the LS-174T i.p. xenograft model

**DOI:** 10.1002/cam4.132

**Published:** 2013-09-19

**Authors:** Kwon J Yong, Diane E Milenic, Kwamena E Baidoo, Young-Seung Kim, Martin W Brechbiel

**Affiliations:** 1 Radioimmune and Inorganic Chemistry Section, Radiation Oncology Branch, National Cancer Institute, National Institutes of HealthBethesda, Maryland

**Keywords:** ^212^Pb-TCMC-trastuzumab, disseminated intraperitoneal disease, gene expression

## Abstract

Recent studies have demonstrated that therapy with ^212^Pb-TCMC-trastuzumab resulted in (1) induction of apoptosis, (2) G2/M arrest, and (3) blockage of double-strand DNA damage repair in LS-174T i.p. (intraperitoneal) xenografts. To further understand the molecular basis of the cell killing efficacy of ^212^Pb-TCMC-trastuzumab, gene expression profiling was performed with LS-174T xenografts 24 h after exposure to ^212^Pb-TCMC-trastuzumab. DNA damage response genes (84) were screened using a quantitative real-time polymerase chain reaction array (qRT-PCR array). Differentially regulated genes were identified following exposure to ^212^Pb-TCMC-trastuzumab. These included genes involved in apoptosis (*ABL*, *GADD45α*, *GADD45γ*, *PCBP4*, and *p73*), cell cycle (*ATM*, *DDIT3*, *GADD45α*, *GTSE1*, *MKK6*, *PCBP4*, and *SESN1*), and damaged DNA binding (DDB) and repair (*ATM* and *BTG2*). The stressful growth arrest conditions provoked by ^212^Pb-TCMC-trastuzumab were found to induce genes involved in apoptosis and cell cycle arrest in the G2/M phase. The expression of genes involved in DDB and single-strand DNA breaks was also enhanced by ^212^Pb-TCMC-trastuzumab while no modulation of genes involved in double-strand break repair was apparent. Furthermore, the p73/GADD45 signaling pathway mediated by p38 kinase signaling may be involved in the cellular response, as evidenced by the enhanced expression of genes and proteins of this pathway. These results further support the previously described cell killing mechanism by ^212^Pb-TCMC-trastuzumab in the same LS-174T i.p. xenograft. Insight into these mechanisms could lead to improved strategies for rational application of radioimmunotherapy using α-particle emitters.

The apoptotic response and associated gene modulations have not been clearly defined following exposure of cells to α-particle radioimmunotherapy (RIT). Gene expression profiling was performed with LS-174T i.p. (intraperitoneal) xenografts after exposure to ^212^Pb-TCMC-trastuzumab. Differentially regulated 22 genes were identified following the stressful growth arrest conditions provoked by ^212^Pb-TCMC-trastuzumab, providing an informative approach toward understanding the molecular basis of tumor biology in response to α-particle radiation and leading to improved strategies for RIT using α-particle emitters. This study provides data which is among the first to describe in detail, the cellular response to α-particle irradiation in vivo.

## Introduction

Alpha particles provoke severe tissue damage and induce clusters of DNA strand breaks that lead to cell death by virtue of their high linear energy transfer (LET of ∼100 KeV/μm) and short range in tissue. Thus, α-emitters such as ^213^Bi and ^212^Bi are superior with respect to cell killing to β^−^-emitters which are low LET and possess long range in tissue. In a study involving α-radiation exposure of cells, the probability of cell death or loss of reproductive capability was 66% per α-traversal of the cell 1. The unique cell killing capabilities of α-radiation make application of immunoconjugates labeled with α-emitter radionuclides a promising therapeutic option for the treatment of patients with carcinomas that are characterized by disseminated single tumor cells in the peritoneum such as ovarian or gastric cancer 2,3. An α-particle emitting source regarded as potentially useful for such therapeutic applications is ^212^Pb, the longer lived parental radionuclide of ^212^Bi for which ^212^Pb serves as an in vivo generator 4.

Exposure of cells to ionizing radiation activates multiple signal transduction pathways, which results in complex alterations in gene expression. The transcriptional responses to genotoxic stresses can be monitored on a global scale using microarray. In addition, specific gene expression can be evaluated with greater accuracy and sensitivity by quantitative real-time polymerase chain reaction (qRT-PCR) 5–7. Such gene expression profiling may provide a powerful approach toward understanding the molecular basis of tumor biology in response to radiation. Studies of gene expression in this manner may lead to the identification of radiation responsive genes that could potentially provide biomarkers of radiation exposure.

A recent study from this laboratory demonstrated that the reduction of cell proliferation elicited by the α-emitting radioimmunotherapeutic, ^212^Pb-TCMC-trastuzumab, is associated with G2/M arrest, blockage of double-strand DNA damage repair and increased apoptosis 5. To date, gene modulations associated with α-particle radioimmunotherapy (RIT) have not been defined 9,10. Therefore, to better understand the molecular basis of α-particle RIT, the changes in gene expression in response to α-emitter RIT were investigated. The aim of this study was to evaluate the gene expression profile associated with the key biological processes of apoptosis, cell cycle arrest, and DNA repair elicited in LS-174T i.p. (intraperitoneal) xenografts after targeted exposure to ^212^Pb-TCMC-trastuzumab. Eighty-four genes involved in DNA damage signaling pathways were quantified using qRT-PCR array 24 h after the ^212^Pb-RIT. This study provides data which is among the first to describe in detail, the cellular response to α-particle irradiation in vivo.

## Materials and Methods

### Cell line

The human colon carcinoma cell line (LS-174T) was used for the in vivo studies and grown in supplemented Dulbecco's modified Eagle's medium (DMEM). All media and supplements were obtained from Lonza (Walkersville, MD). The cell line has been screened for mycoplasma and other pathogens before in vivo use according to the NCI Laboratory Animal Sciences Program policy. No authentication of the cell line was conducted by the authors.

### Chelate synthesis, mAb conjugation, and radiolabeling

The synthesis, characterization, and purification of the bifunctional ligand TCMC have been previously described 3. Trastuzumab (Herceptin®; Genentech, South San Francisco, CA) was conjugated with TCMC by established methods using a 10-fold molar excess of ligand to mAb and radiolabeled as detailed elsewhere 3,11. A 10 mCi ^224^Ra/^212^Pb generator was purchased from AlphaMed (Lakewood, NJ). Purified polyclonal human IgG (HuIgG) fraction (ICN, Irvine, CA) was similarly conjugated with the TCMC ligand and radiolabeled with ^212^Pb as described above, providing a nonspecific control antibody for the experiments.

### Tumor model, treatment, and tumor harvesting

Studies were performed with 19–21 g female athymic mice (NCI-Frederick) bearing 3 day i.p. LS-174T xenografts as previously reported 3. The viability of the LS-174T cells (>95%) was determined using trypan blue. Mice were injected i.p. with 1 × 10^8^ LS-174T cells in 1 mL of DMEM. The inoculum size for this cell line represented the minimum number of cells required for tumor growth in 100% of the mice. ^212^Pb-TCMC-trastuzumab (10 μCi in 0.5 mL phosphate buffered saline [PBS]) was administered to the mice 3 days postimplantation of tumor (*n* = 10–15). This treatment group was compared with sets of tumor-bearing mice that received ^212^Pb-TCMC-HuIgG, unlabeled trastuzumab or HuIgG, or no treatment. Mice receiving trastuzumab or HuIgG were injected 3 day after tumor implantation with 10 μg of the respective material. Tumors were harvested from mice bearing i.p. LS-174T xenografts at 24 h post-treatment. Tumor tissues were stored at −80°C until use. All animal protocols were approved by the National Cancer Institute Animal Care and Use Committee.

### RNA purification

Total RNA was isolated from tumor tissues using the RNeasy mini kit (Qiagen, Santa Clarita, CA) according to the manufacturer's instruction and stored at −80°C until use for gene expression qRT-PCR arrays. Purity of the isolated total RNA was measured using Nano-drop and PCR with β-actin primers. Only total RNA with an A260/A280 ratio >1.9 and no detectable contamination of DNA (PCR) was employed for the gene expression array (qRT-PCR array).

### Human DNA damage PCR array

The cDNA was reverse transcribed from RNA using the First Strand cDNA Synthesis Kit (SABiosciences, Frederick, MD). Comparison of the relative expression of 84 DNA damage-related genes was characterized with the human DNA damage signaling pathway PCR array (SABiosciences) and the RT^2^ real-time SYBR Green/Rox PCR master mix (SABiosciences) on a 7500 real-time PCR system (Applied Biosystems, Rockville, MD). The array includes genes involved in apoptosis, cell cycle and DDB and repair (Table S3).

### Immunoblotting

Immunoblot analysis following standard procedures was performed with total protein isolates using tissue protein extraction reagent (T-PER) (ThermoFisher Scientific, Asheville, NC) containing protease inhibitors (Roche, Indianapolis, IN). Fifty microgram of total protein per lane was separated on a 4–20% tris-glycine gel and transferred to a nitrocellulose membrane. Antibodies against phospho-p73, Gadd45α, pMKK6, and pMKK4 (Cell Signaling, Beverly, MA), and Gadd45γ (SantaCruz, Santa Cruze, CA) were used at a dilution of 1:1000 in PBS containing 5% bovine serum albumin (BSA) and 0.05% Tween-20. Horseradish peroxidase-conjugated rabbit secondary antibodies were used at 1:5000 in 3% nonfat dry milk. The blots were developed using the ECL Plus chemoluminescent detection kit (GE Healthcare, Pascataway, NJ) and the images acquired using a Fuji LAS 4000 imager (Fujifilm, Stanford, CT).

## Results

PCR array was used for analyzing gene expression in three independent experiments. The array identified genes that were significantly up- or downregulated 24 h after the ^212^Pb-TCMC-trastuzumab therapy. All experimental group results, that is, labeled antibody, labeled control HuIgG, and unlabeled antibody were compared to harvested untreated tumor and a threshold of a twofold change was applied.

### ^212^Pb-TCMC-trastuzumab-induced cell killing is associated with upregulation of genes involved in apoptosis

Changes in expression of responsive genes involved in apoptosis upon treatment of the LS-174T tumor xenografts with ^212^Pb-TCMC-trastuzumab and ^212^Pb-TCMC-HuIgG as well as to unlabeled HuIgG and trastuzumab are presented in Table 1. Of the 84 genes examined, 13 genes are involved in regulation of the apoptotic process. Seven of the 13 genes (*ABL*, *CIDEA*, *GADD45α*, *GADD45γ*, *IP6K3*, *PCBP4*, and *p73*) appeared to be upregulated following the treatment with ^212^Pb-TCMC-trastuzumab. Five of the gene changes (*ABL*, *GADD45α*, *GADD45γ*, *PCBP4*, and *p73*) were significant (*P *<* *0.05). No significant downregulation of genes involved in the apoptotic process was observed. Remarkably, ^212^Pb-TCMC-trastuzumab had a dramatic impact (8.3-fold increase) on the expression of *p73*, a tumor suppressor gene belonging to the p53 family of transcription factors, and on the stress-response family of *GADD45* including *GADD45α* (4.1-fold increase) and *GADD45γ* (10.3-fold increase). In contrast, ^212^Pb-TCMC-HuIgG, trastuzumab, and HuIgG resulted in the upregulation of only the *GADD45γ* gene with a 3.5-, 2.9-, and 3.2-fold increase, respectively. The *p73* gene seemed to be downregulated in these same groups, though not significantly except trastuzumab treatment alone. Taken together, the increased stressful growth arrest conditions imposed by ^212^Pb-TCMC-trastuzumab induced cell death-associated genes, evidenced by the upregulated expression of genes, including the *GADD45* family and *p73* involved in regulation of the apoptotic processes.

**Table:** Genes expression involved in apoptosis in LS-174T i.p. xenografts by ^212^Pb-TCMC-trastuzumab therapy

Symbol	GeneBank ID	Fold change									
		^212^Pb-trastuzumab	*P-*value	^212^Pb-HuIgG	*P*-value	Trastuzumab	*P*-value	HuIgG	*P*-value
ABL	NM_005157	2.7	0.040	1.1	0.040	1.1	0.119	−1.6	0.226
CIDEA	NM_001279	4.9	0.125	1.9	0.002	−1.1	0.690	1.1	0.894
GADD45α	NM_001924	4.1	0.003	1.4	0.352	1.2	0.490	−1.2	0.940
GADD45γ	NM_006705	10.3	0.037	3.5	0.072	2.9	0.028	3.2	0.025
IP6K3	NM_054111	5.8	0.327	1.5	0.102	−1.9	0.035	−1.0	0.883
PCBP4	NM_020418	2.8	0.026	1.3	0.317	1.2	0.302	−1.0	0.950
p73	NM_005427	8.3	0.039	−1.1	0.578	−2.2	0.009	−1.1	0.850

Mice-bearing i.p. LS-174T xenografts were treated with ^212^Pb-TCMC-trastuzumab for 24 h. qRT-PCR array was used for gene expression analysis in three independent experiments. The numbers indicate fold change (≥twofold) compared to the untreated control. Additional groups include nonspecifically targeted control, ^212^Pb-TCMC-HuIgG, and unlabeled HuIgG, as well as trastuzumab alone. Results represent the average of a minimum of three replicates.

### ^212^Pb-TCMC-trastuzumab induces genes involved in G2/M arrest

Gene expression profiling was examined for genes involved in the regulation of cell cycle arrest (15 genes) and the cell cycle checkpoint (eight genes). Significant differences (>twofold, *P *<* *0.05) in expression were found for seven genes, upregulation of five (*ATM*, *GADD45α*, map kinase kinase 6 [*MKK6*], *PCBP4*, and *SESN1*) and downregulation of two (*DDIT3* and *GTSE1*) after exposure to ^212^Pb-TCMC-trastuzumab (Table 2). Upregulation of *ZAK* did not appear to be significant. The nonspecific control, ^212^Pb-TCMC-HuIgG, upregulated *MKK6* and *SESN1* and downregulated *DDIT3* while trastuzumab alone upregulated *MKK6* and *SESN1* but *SESN1* upregulation was not significant. Among those genes identified, *GADD45α* was upregulated (4.1-fold increase) and *GTSE1* was markedly downregulated (7.5-fold decrease) by ^212^Pb-TCMC-trastuzumab. In contrast, genes involved in cell cycle checkpoint were not significantly modulated in any of the treatment groups. These data suggest that the ^212^Pb-TCMC-trastuzumab treatment suppresses cell proliferation by inducing genes which are involved in cell cycle arrest in G2/M.

**Table:** Genes expression involved in cell cycle in LS-174T i.p. xenografts by ^212^Pb-TCMC-trastuzumab therapy

Symbol	GeneBank ID	Fold change									
		^212^Pb-trastuzumab	*P*-value	^212^Pb-HuIgG	*P*-value	Trastuzumab	*P*-value	HuIgG	*P*-value
ATM	NM_000051	2.6	0.049	−1.5	0.213	−1.4	0.318	−2.2	0.127
DDIT3	NM_004083	−2.4	0.002	−3.5	0.001	−1.8	0.074	−1.6	0.162
GADD45α	NM_001924	4.1	0.003	1.4	0.252	1.2	0.493	−1.2	0.940
GTSE1	NM_016426	−7.5	0.001	−1.7	0.144	−1.4	0.144	−1.1	0.478
MKK6	NM_002758	2.8	0.016	2.6	0.003	2.3	0.005	1.9	0.077
PCBP4	NM_020418	2.7	0.026	1.3	0.317	1.2	0.302	−1.2	0.959
SESN1	NM_014454	2.3	0.001	2.6	0.002	2.0	0.125	1.3	0.227
ZAK	NM_016653	2.1	0.355	1.2	0.104	1.2	0.135	1.4	0.056

Mice-bearing i.p. LS-174T xenografts were treated with ^212^Pb-TCMC-trastuzumab for 24 h. qRT-PCR array was used for gene expression analysis in three independent experiments. The numbers indicate fold change (≥twofold) compared to the untreated control. Additional groups include nonspecifically targeted control, ^212^Pb-TCMC-HuIgG, and unlabeled HuIgG, as well as trastuzumab alone. Results represent the average of a minimum of three replicates.

### ^212^Pb-TCMC-trastuzumab-induced cell killing is associated with genes which are involved in DDB and repair

Twenty-four hours after α-irradiation with ^212^Pb-TCMC-trastuzumab qRT-PCR was next utilized to identify genes involved in DDB (26 genes), single-strand break repair (including nucleotide excision repair (NER; 12 genes), base-excision repair (BER; seven genes), mismatch repair (MR; 14 genes), and double-strand break repair (DSB; nine genes). Among those genes involved in DDB, ten genes (*ATM*, *ATRX*, *BTG2*, *DDB1*, *ERCC1*, *ERCC2*, *SEMA4*, *XPC*, and *XRCC3* [Table 3], *p73* [Table 1]) seemed to be upregulated. Of these only three (*ATM*, *BTG2*, and *p73*) were significantly upregulated. *Cry1* was the only gene downregulated after ^212^Pb-TCMC-trastuzumab therapy. In contrast, ^212^Pb-TCMC-HuIgG resulted in the upregulation of *BTG2* and *XPC* and trastuzumab alone borderline upregulated *BTG2*. *p73* and *GADD45* are upregulated by MR-dependent *ABL*. It has been also shown that MR-dependent intrinsic apoptosis is p53-independent but stimulated by MLH1/ABL/p73/GADD45 retrograde signaling 12. *p73*, which is involved in MR was the only gene in this category that was upregulated after ^212^Pb-TCMC-trastuzumab therapy with a calculated 8.3-fold increase compared to 1.1-fold decrease after ^212^Pb-TCMC-HuIgG therapy. In NER, *DDB1*, *ERCC1*, *ERCC2*, and *XPC* were seemingly upregulated albeit nonsignificantly, while in BER, no genes were found to be differentially expressed in any of the treatment groups. Among those genes related to DNA repair, *BTG2* (7.0-fold increase) was markedly upregulated while *Cry1* (3.3-fold decrease) was downregulated by treatment with ^212^Pb-TCMC-trastuzumab. The downregulation of Cry1, however, did not reach statistical significance. In contrast, ^212^Pb-TCMC-HuIgG resulted in the upregulation of *BTG2* (3.7-fold increase) and downregulation of *Cry1* (1.0-fold decrease). Interestingly, among the genes identified in the profile, no genes related to DSB repair were differentially expressed.

**Table:** Genes expression involved in DNA repair in LS-174T i.p. xenografts by ^212^Pb-TCMC-trastuzumab therapy

Symbol	GeneBank ID	Fold change									
		^212^Pb-trastuzumab	*P*-value	^212^Pb-HuIgG	*P*-value	Trastuzumab	*P*-value	HuIgG	*P*-value
ATM	NM_000051	2.6	0.049	−1.5	0.803	−1.4	0.818	−2.1	0.127
ATRX	NM_000489	2.6	0.137	1.6	0.033	1.4	0.087	−1.2	0.373
BTG2	NM_006763	7.0	0.005	3.7	0.003	1.9	0.003	1.4	0.269
CRY1	NM_004075	−3.3	0.941	−1.0	0.573	1.2	0.157	1.1	0.426
DDB1	NM_001923	2.0	0.319	1.0	0.974	1.4	0.050	1.7	0.205
ERCC1	NM_001983	3.0	0.157	−1.2	0.605	−1.0	0.270	1.4	0.156
ERCC2	NM_000400	2.7	0.209	1.4	0.001	1.6	0.704	1.0	0.588
SEMA4A	NM_022367	4.7	0.186	1.6	0.036	−1.3	0.160	−1.1	0.989
XPC	NM_004628	3.3	0.058	2.3	0.042	1.1	0.028	−1.3	0.712
XRCC3	NM_005432	2.7	0.378	−1.5	0.380	1.4	0.339	−1.2	0.709

Mice-bearing i.p. LS-174T xenografts were treated with ^212^Pb-TCMC-trastuzumab for 24 h. qRT-PCR array was used for gene expression analysis in three independent experiments. The numbers indicate fold change (≥twofold) compared to the untreated control. Additional groups include nonspecifically targeted control, ^212^Pb-TCMC-HuIgG, and unlabeled HuIgG, as well as trastuzumab alone. Results represent the average of a minimum of three replicates.

### ^212^Pb-TCMC-trastuzumab induces cell cycle arrest and apoptosis by the p73/GADD45 signaling pathway

**Figure 1:**
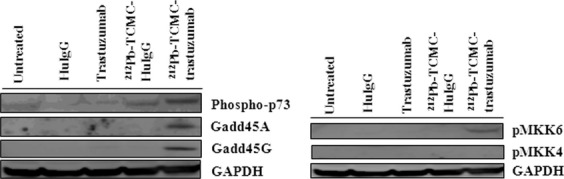
p73/GADD45 signaling is activated in response to ^212^Pb-TCMC-trastuzumab. Immunoblot analysis for p73, GADD45, MKK6, and MKK4 was performed with tumors collected 24 h after ^212^Pb-TCMC-trastuzumab treatment. Phospho-p73 was detected at 80 kDa; GADD45α and γ were visualized at 22 kDa. MKK6 and MKK4 were detected at 41 and 44 kDa, respectively. GAPDH was served as the equal protein loading control.

Many genes involved in cell cycle arrest and apoptosis are regulated at the transcriptional level by *p73* 13. *GADD45* is a well-defined downstream gene of *p73* and *p53*, and is activated by *p73*. *GADD45* has critical roles in negative cell growth control and apoptosis 14,15. In response to a DNA damage signal, activated ABL kinase induces phosphorylation of p73 and the p38 mitogen-activated protein (MAP) kinase pathway mediates this response 16. ^212^Pb-TCMC-trastuzumab increased the expression of *ABL*, *p73*, and the *GADD45* gene family compared to the nonspecific control or trastuzumab alone. To investigate the role of *p73/GADD45*-induced cell cycle arrest and apoptosis in LS-174T i.p. xenografts from mice treated with ^212^Pb-TCMC-trastuzumab, the expression of *p73* and *GADD45* at the protein level was first determined using immunoblots. Figure 1 shows that phosphorylation of p73 was enhanced by ^212^Pb-TCMC-trastuzumab treatment compared to the controls. Expression of the *GADD45* genes, downstream of *p73*, was also enhanced in tumors treated with ^212^Pb-TCMC-trastuzumab, indicating that ^212^Pb-TCMC-trastuzumab increased the expression of genes involved in apoptosis and cell cycle arrest at both the transcriptional and protein level.

p38 activation is required for the ability of ABL to induce apoptosis, and p38 MAP kinase is required for the activation of p73 by ABL. Activation of MKK6/p38 is sufficient to increase p73 protein levels 16. The GADD45 family, GADD45α and GADD45γ, have been found to utilize JNK/p38 pathways, leading to G2/M cell cycle arrest 17. Based on gene profiles induced by ^212^Pb-TCMC-trastuzumab, *MKK6*, an activator of p38, which is required for ABL to induce apoptosis, was significantly upregulated. To examine whether activation of *p73/GADD45* induced by ^212^Pb-TCMC-trastuzumab is mediated through JNK/p38 pathways, the expression of *MKK6* and map kinase kinase 4 (*MKK4*) at the protein levels were determined using Western blot analysis. The results showed that ^212^Pb-TCMC-trastuzumab enhanced phosphorylation of MKK6, an activator of p38, but phosphorylation of MKK4, an activator of JNK was not observed (Fig. 1), suggesting that the stressful growth arrest conditions by ^212^Pb-TCMC-trastuzumab may be mediated by the p38 signaling pathway.

## Discussion

Exposure to radiation leads to complex cellular responses that include changes in gene expression. This behavior appears to be different between low LET γ- or β^−^-irradiation and high LET α-particle irradiation. The rate of DNA repair in damaged cells after exposure to high LET α-irradiation has been shown to be much slower than the rate observed in cells exposed to low LET irradiation. This is attributed to the ability of α-particles to induce multiple ionizations within the DNA structure and in adjacent molecules, resulting in severe locally damaged sites that are less likely to be repaired 4,18. This renders α-radiation highly efficient in cell killing. Incorporation of α-emitters in radioimmunotherapeutics attempts to specifically target α-radiation to pathologies in tissue to take advantage of this exquisite cell killing property. Toward this end, the therapeutic efficacy of ^212^Pb-TCMC-trastuzumab, an α-emitting radioimmunotherapeutic, given as a single injection has been demonstrated 3. To better understand the molecular basis of ^212^Pb-TCMC-trastuzumab-induced cell death in vivo, gene expression profiling was performed to screen for genes affected by ^212^Pb-TCMC-trastuzumab in LS-147T i.p. xenografts. The primary target of ionizing radiation in tissue is DNA and the biological responses to the DNA damage signal are complex. Thus, the experimental approach utilized in this study can only hope to identify major pathways likely to dominate the response.

Altogether, 84 genes involved in DNA damage signaling pathways were analyzed by a qRT-PCR array. The genes analyzed here are associated with the ATR/ATM (ataxia telangiectasia mutated) signaling network and transcriptional targets of the DNA damage response. The differentially expressed genes were functionally classified into biological categories (Table S3). Among them, a set of 22 genes were identified that seemed to be differentially expressed 24 h after administration of ^212^Pb-TCMC-trastuzumab (Tables S1, S2). The level of differential expression of half of these genes reached statistical significance. Many of the upregulated genes identified in this study are known to play key roles in the DNA damage response, apoptosis, and cell cycle arrest. These include *ABL*, *ATM*, *p73*, and the *GADD45* family. The findings support the recent study from this laboratory that demonstrated that ^212^Pb-TCMC-trastuzumab induces G2/M arrest and apoptosis, in vivo, in the colon cancer LS-174T i.p. xenograft model 8. The upregulated genes identified in these experiments could favor confinement of the DNA damage by delaying initiation of the cell cycle, leading to induction of apoptosis as a result of ^212^Pb-TCMC-trastuzumab therapy. As indicated in the results section, *p73* appears to be involved in all three major biological responses to DNA damage, namely, DNA damage repair (MR), apoptosis, and cell cycle arrest.

Given the upregulated genes identified, it was hypothesized that ^212^Pb-TCMC-trastuzumab may induce G2/M arrest and apoptosis through the p73/GADD45 signaling pathway, which might be mediated by p38 kinase signaling. As indicated in Tables 1 and 2, both the ABL and ATM genes were found to be significantly upregulated. ATM is activated in response to DNA damage and signals the presence of DNA damage by phosphorylating targets involved in cell cycle arrest and DNA repair. In mammals, ATM functions as a critical regulator of the cellular DNA damage response. ABL is a nonreceptor tyrosine kinase that is potently activated in DNA damage and is one of several key molecules, including ATM, DNAPK, and p53, involved in coordinating the response to DNA insults 19–22. In response to a DNA damage signal, activated ABL kinase induces phosphorylation of p73 and the p38 MAP kinase pathway mediates this response 14. ABL has been known to stimulate JNK and p38 kinase and to play a central role in the apoptotic response to DNA damage 23,24. ABL/GADD45 control G2 arrest and apoptosis, whereas ABL/p73 is needed in apoptosis and survival. *p73*, a member of the tumor suppressor *p53* family, is similar to *p53* both functionally and structurally, and induces cell cycle arrest and cell death in response to DNA damage. *p73* can activate the promoters of several p53 response genes including *p21*, *BAX*, *MDM2*, *GADD45*, and *IGFBP3* 13,23.

The *GADD45* (growth arrest and DNA damage-inducible 45) gene family encodes three proteins, GADD45α, β, and γ. GADD45 proteins functions are similar, but not identical and their induction differs under diverse physical conditions or in different cell types. The GADD45 family is p73/p53-regulated, a DNA damage-inducible biomarker and a negative growth-control gene that has also been found to be an important player in the DNA damage response, causing cell cycle arrest at the G2/M phase. GADD45 is also known to interact with key cell regulators such as p21, CDC2/CYCLIN B1, PCNA, p38, and MEEK4 14,15. The *GADD45* genes are rapidly regulated by *p53* which may play a role in apoptosis by activating the JNK and/or p38 mitogen-activated protein kinase (MAPK) signaling pathways 24.

**Figure 2:**
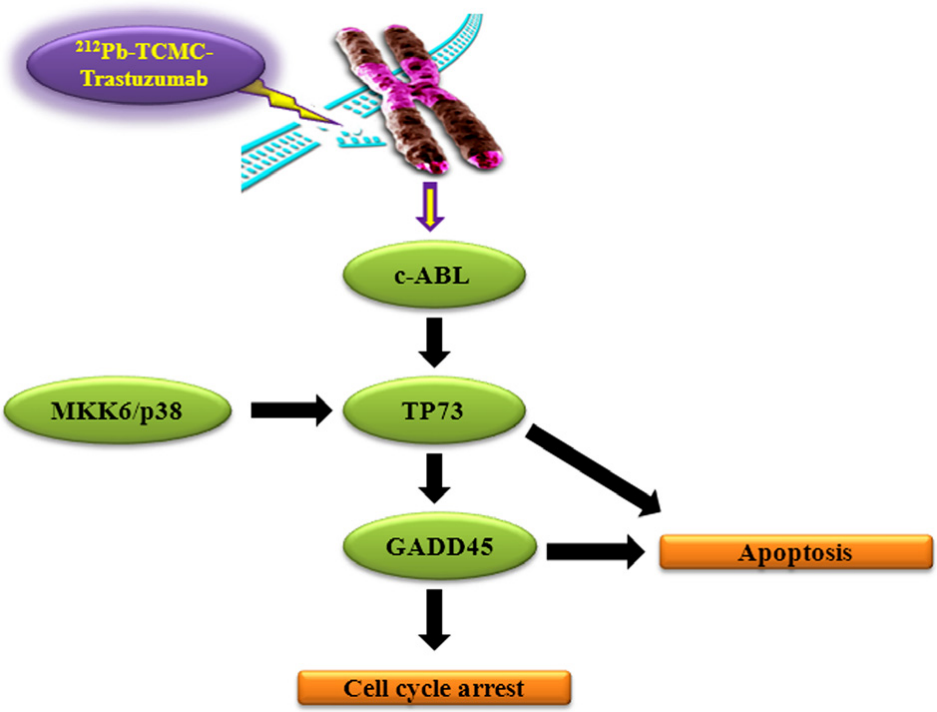
p73/GADD45 signaling induced by ^212^Pb-TCMC-trastuzumab leads to apoptosis and cell cycle arrest. See text for details.

Figure 1 shows that ^212^Pb-TCMC-trastuzumab treatment induced phosphorylation of p73 and expression of the *GADD45* family at the protein level, as evidenced by immunoblot analysis, suggesting that the p73/GADD45 signaling pathway is activated in response to ^212^Pb-TCMC-trastuzumab. Recently, *p73* has been found to induce the JNK apoptotic signaling pathway in response to cisplatin in ovarian cancer cells 25. The induction of apoptosis was shown to be dependent upon the interaction of GADD45 protein with MKK4, an upstream activator of stress-induced p38/JNK kinases. GADD45α and GADD45γ have also been reported to utilize both the p38 and JNK pathways to induce G2M arrest 17. Figure 1 also shows that ^212^Pb-TCMC-trastuzumab therapy results in the phosphorylation of MKK6 (p38kinase activator) and not MKK4 (JNK kinase activator), suggesting that activation of p73 induced by ^212^Pb-TCMC-trastuzumab may be mediated by p38 kinase signaling. In response to γ-irradiation, p38 MAPK is an ATM-dependent event. MKK6 (MAP2K6, mitogen-activated protein kinase kinase 6) is involved in many cellular processes such as stress-induced cell cycle arrest, transcription activation, and apoptosis. The MKK6-p38 cascade forms an essential part of the signaling pathway leading to G2 arrest 26. Although *GADD45*, *IP6K3* (inositol hexakisphosphate kinase 3), and *PCBP4* (Poly(rC)-binding protein 4) have been known to induce apoptosis mediated by *p53*, activation of *p53* was not observed after ^212^Pb-TCMC-trastuzumab treatment. Apoptotic pathways triggered by high LET radiation do not require p53. The activation of p73 mediated by p38 may, therefore, play an important role in the initiation of apoptotic programs and cell cycle arrest in response to ^212^Pb-TCMC-trastuzumab in many tumors that lack a functional p53, but do express p73 (Fig. 2).

Cells cannot function if DNA damage corrupts the integrity and accessibility of essential information in the genome. Genes related to DNA repair that were identified to be deferentially expressed significantly include *ATM* and *BTG2*. *BTG2* (BTG family member 2) function may be related to cell cycle control and cellular response to DNA damage 27. Interestingly, no modulation of genes involved in DSB repair was apparent while the expression of genes involved in DDB and single-strand DNA breaks were enhanced by ^212^Pb-TCMC-trastuzumab. These results suggest that ^212^Pb-TCMC-trastuzumab treatment would result in inefficient DSB repair at a time when a high degree of DSBs have occurred as a result of the targeted α-irradiation. Severe DNA DSBs caused by α-emitting radiation have been known to be inefficiently repaired, leading to cell death 4.

In conclusion, multiple genes were identified in the colon cancer LS-174T i.p. xenograft model used by this laboratory that responded to ^212^Pb-TCMC-trastuzumab therapy. Modulation of such genes points to their potential role in apoptosis, cell cycle, and DNA repair after exposure to acute high LET radiation. These genes could provide a potent research tool for examination of the therapeutic responses to and mechanisms of α-particle RIT for the treatment and management of patients with disseminated peritoneal disease. Identifying genes pivotal in patients' responses may also provide direction for optimizing combination therapies. Insight into these mechanisms could lead to improved strategies for RIT with α-emitters and promote the clinical translation of such agents.
