# Supplementary material for: Gene expression profiling upon 212Pb-TCMC-trastuzumab treatment in the LS-174T i.p. xenograft model
Source: Cancer Med. 2013 Sep 19;2(5):646–53. doi: 10.1002/cam4.132 (PMC3892796; doi:10.1002/cam4.132)
Supplement: Supplementary file 1 [file cam40002-0646-sd1.doc]

**Table S1**. Up-regulated genes induced by ^212^Pb-TCMC-trastuzumab in LS-147T i.p. xenografts.

| **Symbol** | **GeneBank ID** | **Fold change**  **^212^Pb-trastuzumab** *p-value* **^212^Pb-HuIgG** *p-value* **Trastuzumab** *p-value* **HuIgG** *p-value* |
| --- | --- | --- |
| ABL | NM_005157 | 2.7 0.040 1.1 0.040 1.1 0.119 -1.6 0.226 |
| ATM | NM_000051 | 2.6 0.049 -1.5 0.213 -1.4 0.318 -2.2 0.127 |
| ATRX | NM_000489 | 2.6 0.137 1.6 0.033 1.4 0.087 -1.2 0.373 |
| BTG2 | NM_006763 | 7.0 0.005 3.7 0.003 1.9 0.003 1.4 0.269 |
| CIDEA | NM_001279 | 4.9 0.125 1.9 0.002 -1.1 0.690 1.0 0.894 |
| DDB1 | NM_001923 | 2.0 0.319 1.0 0.974 1.4 0.050 1.7 0.205 |
| ERCC1 | NM_001983 | 3.0 0.157 -1.2 0.605 -1.0 0.270 1.4 0.156 |
| ERCC2 | NM_000400 | 2.7 0.209 1.4 0.001 1.6 0.704 1.0 0.588 |
| GADD45α | NM_001924 | 4.1 0.003 1.4 0.352 1.2 0.490 -1.2 0.940 |
| GADD45γ | NM_006705 | 10.3 0.037 3.5 0.072 2.9 0.028 3.2 0.025 |
| IP6K3 | NM_054111 | 5.8 0.327 1.5 0.102 -1.9 0.035 -1.0 0.883 |
| MKK6 | NM_002758 | 2.8 0.016 2.6 0.003 2.3 0.005 1.9 0.077 |
| PCBP4 | NM_020418 | 2.7 0.026 1.3 0.317 1.2 0.302 -1.2 0.959 |
| SEMA4A | NM_022367 | 4.7 0.186 1.6 0.036 -1.3 0.160 -1.1 0.989 |
| SESN1 | NM_014454 | 2.3 0.001 2.6 0.002 2.0 0.125 1.3 0.227 |
| p73 | NM_005427 | 8.3 0.039 -1.1 0.578 -2.2 0.009 -1.1 0.850 |
| XPC | NM_004628 | 3.3 0.058 2.3 0.042 1.1 0.028 -1.3 0.712 |
| XRCC3 | NM_005432 | 2.7 0.378 -1.5 0.380 1.4 0.339 -1.2 0.709 |
| ZAK | NM_016653 | 2.1 0.355 1.2 0.104 1.2 0.135 1.4 0.056 |
