# Supplementary material for: Gene expression profiling upon 212Pb-TCMC-trastuzumab treatment in the LS-174T i.p. xenograft model
Source: Cancer Med. 2013 Sep 19;2(5):646–53. doi: 10.1002/cam4.132 (PMC3892796; doi:10.1002/cam4.132)
Supplement: Supplementary file 2 [file cam40002-0646-sd2.doc]

**Table S2**. Down-regulated genes induced by ^212^Pb-TCMC-trastuzumab in LS-147T i.p. xenografts.

| **Symbol** | **GeneBank ID** | **Fold change**  **^212^Pb-trastuzumab** *p-value* **^212^Pb-HuIgG** *p-value* **Trastuzumab** *p-value* **HuIgG** *p-value* |
| --- | --- | --- |
| CRY1 | NM_004075 | -3.3 0.941 -1.0 0.573 1.2 0.157 1.1 0.426 |
| DDIT3 | NM_004083 | -2.4 0.002 -3.5 0.001 -1.8 0.074 -1.6 0.162 |
| GTSE1 | NM_016426 | -7.5 0.001 -1.7 0.144 -1.4 0.144 -1.1 0.478 |
