# Supplementary material for: Gene expression profiling upon 212Pb-TCMC-trastuzumab treatment in the LS-174T i.p. xenograft model
Source: Cancer Med. 2013 Sep 19;2(5):646–53. doi: 10.1002/cam4.132 (PMC3892796; doi:10.1002/cam4.132)
Supplement: Supplementary file 3 [file cam40002-0646-sd3.doc]

Table S3. Functional gene grouping

**APOPTOSIS**: ABL, BRCA1, CIDIA, GADD45α, GADD45γ, GML, IP6K3, PCBP4, AIFM1 (PDCD8), PPP1R15A, RAD21, p53, p73

**CELL CYCLE ARREST**: CHEK1, CHEK2, DDIT3 (CHOP), GADD45α, GML, GTSE1, HUS1, MKK6, MAPK12, PCBP4, PPP1R15A, RAD17, RAD9A, SESN1, ZAK

**CELL CYCLE CHECK POINT**: ATR, BRCA1, FANCG, NBN (NBS1), RAD1, RBBBP8, SMC1A (SMC1L1), p53

**DAMAGED DNA BINDING**: ANKRD17, BRCA1, DDB1, DMC1, ERCC1, FANCG, FEN1, MPG, MSH2, MSH3, N4BP2, NBN (NBS1), OGG1, PMS2P3 (PMS2L9), PNKP, RAD1, RAD18, RAD51, RAD51B, REV1 (REV1L). SEMA4A, XPA, XPC, XRCC1, XRCC2, XRCC3

**NUCLEOTIDE-EXCISION REPAIR**: DDB1, ERCC1, ERCC2 (XPD), LIG1, NTHL1, OGG1, PCNA, PNKP, RPA1, p53, XPA, XPC

**BASE-EXCISION REPAIR**: APEX1, MBD4, MPG, MUTYH, NTHL1, OGG1, UNG

**MISMATCH REPAIR**: ABL1, ANKRD17, EXO1, MLH1, MLH3, MSH2, MSH3, MUTYH, N4BP2, PMS1, PMS2, PMS2P3 (PMS2L9), p73, TREX1

**DOUBLE STRAND BREAK REPAIR**: CIB1, FEN1, XRCC6 (G22P1), XRCC6BP1 (KUB3), MRE11A, MNN (NBS1), PRKDC, RAD21, RAD50

**OTHER GENES RELATED TO DNA REPAIR**: ATM, ATRX, BTG2, CCNH, CDK7, CRY1, ERCC2 (XPD), GTF2H1, GTF2H2, IGHMBP2, LIG1, MNAT1, PCNA, RPA1, SUMO1
