# Supplementary material for: Gene expression profiling upon 212Pb-TCMC-trastuzumab treatment in the LS-174T i.p. xenograft model
Source: Cancer Med. 2013 Sep 19;2(5):646–53. doi: 10.1002/cam4.132 (PMC3892796; doi:10.1002/cam4.132)
Supplement: Supplementary file 4 [file cam40002-0646-sd4.doc]

| **Symbol**  **Table S4**. Gene symbols and name | **Gene name** |
| --- | --- |
| ABL | c-abl oncogene 1, non-receptor tyrosine kinase |
| ATM | Ataxia telangiectasia mutated |
| ATRX | Alpha thalassemia/mental retardation syndrome X-linked |
| BTG2 | BTG family, member 2 |
| CIDEA | Cell death-inducing DEFA-like effector a |
| DDB1 | Damage-specific DNA binding protein 1 |
| ERCC1 | Excision repair cross-completing rodent repair deficiency, complementation group 1 |
| ERCC2 | Excision repair cross-completing rodent repair deficiency, complementation group 2 |
| GADD45α | Growth arrest and DNA-damage-inducible, alpha |
| GADD45γ | Growth arrest and DNA-damage-inducible, gamma |
| IP6K3 | Inositol hexakisphosphate kinase 3 |
| MKK6 | MAP2K6, Mitogen-activated protein kinase kinase 6 |
| PCBP4 | Poly(rC) binding protein 4 |
| SEMA4A | Sema domain, immunoglobulin domain(Ig), transmembrane domain ™ and short cytoplasmic domain, (semaphoring) A |
| SESN1 | Sestrin 1 |
| p73 | Tumor protein p73 |
| XPC | Xeroderma pigmentosum, complementation group C |
| XRCC3 | X-ray repair complementing defective in Chinese hamster cells 3 |
| ZAK | Sterile alpha motif and leucine zipper containing kinase |
| CRY1 | Cryptochrome1 (photolyase-like) |
| DDIT3 | DNA-damage-inducible transcript 3 |
| GTSE1 | G and S-phase expressed 1 |
|  |  |
